# Seroprevalence of measles antibodies and factors associated with susceptibility: a national survey in Mexico using a plaque reduction neutralization test

**DOI:** 10.1038/s41598-020-73618-8

**Published:** 2020-10-15

**Authors:** José Luis Díaz-Ortega, Elizabeth Ferreira-Guerrero, Luis Pablo Cruz-Hervert, Guadalupe Delgado-Sánchez, Leticia Ferreyra-Reyes, Mercedes Yanes-Lane, Norma Mongua-Rodríguez, Rogelio Montero-Campos, Deyanira Castañeda-Desales, Lourdes García-García

**Affiliations:** Centro de Investigación Sobre Enfermedades Infecciosas, Instituto Nacional de Salud Pública, Av. Universidad # 655, Col. Sta. María Ahuacatitlán, C.P. 62100 Cuernavaca, Morelos México

**Keywords:** Viral infection, Preventive medicine, Health policy, Epidemiology

## Abstract

Measles continues to be one of the leading causes of child mortality worldwide, even though a highly effective vaccine has existed for more than 40 years. We aimed to describe the seroprevalence of measles antibodies in Mexico in 2012 and the risk factors associated with susceptibility. A total of 7,785 serum samples were analyzed from the National Health and Nutrition Survey in Mexico. This national survey is representative of the general population, including noninstitutionalized adult, adolescent, and child populations. Antibody titers were classified into protective (> 120 mIU/mL) or susceptible (≤ 120 mIU/mL) levels. The weighted seroprevalence and susceptibility of the overall population were 99.37% (95% CI 99.07–99.58) and 0.63% (95% CI 0.42–0.93), respectively. Among 1-to-4-year-old children, 2.18% (95% CI 1.36–3.48) were susceptible to measles. Among adolescents and young adults, the prevalence of susceptibility was as follows: those 15–19 years of age had a prevalence of 0.22% (95% CI 0.09–0.57), and those 30–39 years of age had a prevalence of 1.17% (95% CI 0.47–2.85). Susceptibility was associated with young age, living in Mexico City, living in crowded households and unknown or nonvaccinated status among 1- to 5-year-old children. Although the overall sample population seroprevalence for measles is above 95%, increased susceptibility among younger children signals the importance of the timely administration of the first vaccine dose at 12 months of age. Furthermore, increased susceptibility among specific subgroups indicates the need to reinforce current vaccination policies, including the immunization of unvaccinated or incompletely vaccinated individuals from 10 to 39 years of age.

## Introduction

Measles is one of the leading causes of child mortality worldwide, even though a highly effective vaccine has existed for more than 40 years. This vaccine ranks among the most cost-effective interventions in public health due to its low cost and its high impact on mortality rates^1^. According to estimates from the World Health Organization (WHO), measles mortality worldwide has decreased by 84% from 2000 to 2016^2,3^.

The WHO Measles and Rubella Global Strategic Plan 2012–2020 established the objective of eliminating measles from the five world regions^4^; presently, all member states have adopted measles elimination goals^5^.

In 2018, based on current trends in measles vaccination coverage and incidence, the WHO Strategic Advisory Group of Experts on Immunization (SAGE) determined that measles elimination is significantly under threat and that the disease has had a resurgence in several countries that had achieved, or were close to achieving, elimination^5^. Endemic measles transmission was re-established in Venezuela in 2018 and in Brazil in 2019^6^. In Mexico, the last case of measles due to endemic transmission occurred in 1995^7^. Between 2000 and February 2020, 373 confirmed cases occurred in Mexico, most of which were imported^7^.

In Mexico, the administration of the monovalent measles vaccine to 12-month-old infants began in 1972. Low vaccination coverage, according to the registry of administered doses, led to a significant measles outbreak with 8,000 deaths and more than 80,000 cases from 1989 to 1990. The national health system introduced the universal administration of the measles, mumps, and rubella (MMR) vaccine to 12-month-old children with a booster dose at age six in 1998. In 2000, the administration of the measles and rubella vaccine was added to the routine schedule among adolescents and adults who had not been previously immunized. In 2008, a national campaign achieved the administration of the measles and rubella vaccine (MR) to all individuals aged 19–29 years old. Presently, the vaccination schedule includes the administration of the MMR vaccine at 12 months of age, a booster dose at age six and the administration of the MR vaccine to individuals from 10 to 39 years of age (if the person has not been vaccinated or has an incomplete vaccination schedule)^8^. As reported to the WHO, the levels of coverage with the first dose of measles-containing vaccine in Mexico in 2014, 2015, 2016 and 2017 were 97%, 97%, 96%, and 96%, respectively^9^.

Serosurveys can provide a direct measure of population immunity and provide high-quality information on the accumulation of susceptible populations, thus contributing to preventing outbreaks. We used serum samples from the 2012 National Health and Nutrition Survey (ENSANUT) in Mexico to estimate the prevalence of antibody titers against measles in children, adolescents, and adults and to identify risk factors associated with susceptibility.

## Material and methods

### Design and study population

The ENSANUT 2012 was a probabilistic, multistage, stratified, cluster household survey conducted by the Government of Mexico's Ministry of Health from August 2011 to June 2012. The research design and details of the sample size and sampling design have been described in detail in the following reference^10^. Briefly, the ENSANUT 2012 included a multistage-stratified selection from each of the 50,528 households visited, and interviews were conducted with a child (1–9 years old), an adolescent (10–19 years old) and an adult (20 years old or older). Blood samples were obtained from 37% of the randomly selected individuals (1 year old and older). The response rate was 87%. Our study consisted of a secondary analysis based on data from 7785 individuals. The sample was obtained by simple random sampling stratified by age selected from the children, adolescents, and adults who had provided a blood sample. This sample size was estimated to provide 80% statistical power at an alpha value of 0.05 and a design effect of 1.7, assuming an overall seroprevalence ranging from 95% (± 2%) in children to 85% (± 2%) in adults and an availability of blood samples or questionnaires between 78.3% and 81.0%.

We obtained the participants’ sociodemographic characteristics and vaccination history from questionnaires that included information on household details, use of public services, and use of health services.

### Laboratory tests

Seven milliliters of venous blood was collected from each participant in Vacutainer SST (Becton–Dickinson) tubes. Samples were transported in refrigerated containers to local laboratories where they were centrifuged for 7 min at 3,000 rpm. The tubes were then stored at 4 °C for no more than five days and transported to the Instituto Nacional de Salud Pública (National Institute of Public Health, INSP) in refrigerated containers. Once at the INSP, aliquots were separated and stored at -190 °C. Measles antibody levels were evaluated by the Plaque Reduction Neutralization Test (PRNT), with > 120 mIU/mL considered to be positive^11–13^. PRNT titers at the time of exposure to the virus have been found to be closely correlated with measles immunity^12^. We performed the technique in two phases, as recommended for large surveys^14^. The first phase was the qualitative PRNT that screened all samples in a 1:4 dilution. All sera that had two or more plaque-forming units (PFUs) were considered negative. We confirmed negative titers with the quantitative PRNT. For the quantitative PRNT, the test serum is diluted six four-fold dilutions, and the diluted samples are then mixed and incubated with a standard inoculum of challenge virus (previous viral titration). Afterwards, a VERO cell suspension is exposed to the mixture of virus and sera, allowing sedimentation to form a monolayer in the microplate. After seven days of incubation, cells were stained with crystal violet, and the PFUs that formed in each of the six wells were counted. We used the World Health Organization anti-measles serum (3rd International Standard (IS) supplied by the National Institute for Biological Standards and Control, South Mimms, United Kingdom) as the reference serum. Edmonston Zagreb was used as the control for the measles virus, with a count between 25 and 35 PFUs per well. The following controls were included in each assay run: (1) we tested the 3rd IS in triplicate using serial four-fold dilutions; (2) we inoculated at least five wells with 25 µL of diluted challenge virus in the absence of serum as a virus control to determine the average plaque count per well; and 3) we inoculated one well with media (without virus or serum) to serve as a cell control. The assay run was considered valid if the average plaque count for the virus control was within the required range of 25 to 35 plaques per well, the cell control well(s) showed no plaques, and the endpoint titer for the reference serum was within the specified limits. Fifty percent endpoint titers (neutralizing dose, ND50) were calculated using the Kärber formula.

### Covariates

Since only children from 1 to 6 years of age had a vaccination card, we considered previous vaccination (having received at least one dose of the MMR vaccine) only for this age group.

We included the following covariates: age and sex of participant; urban/rural residence; household materials; availability of public services; access to social security (yes vs no); region (Northern, Central, Southern and Mexico City); and crowding (yes vs no). The categorization of regions was the same as in previous large national surveys. This regionalization is meaningful for public health officers since it describes areas that have different socioeconomic, demographic, and health care characteristics (more affluent in the Northern region, moderately affluent in the Central region and Mexico City, and not affluent in the Southern region). Although routinely scheduled vaccines are provided free of charge, access to social security is relevant since beneficiaries usually have better access to vaccination services. We used the same classification of urban (≥ 2,500 inhabitants) and rural (< 2,500 inhabitants) localities as that used by the Instituto Nacional de Estadística y Geografía (National Institute of Geography and Statistics, INEGI). This denomination does not include an extension. We used a standard socioeconomic index developed in Mexico based on various household characteristics, including building materials, the number of rooms, basic service infrastructure, and ownership of domestic appliances. This index was selected to allow comparison with previous surveys in Mexico^15^.

### Statistical analysis

We calculated the prevalence and 95% confidence intervals (95% CI) of antibodies for titers ≤ 120 mIU/mL and > 120 mIU/mL stratified by previous vaccination status and demographic and socioeconomic variables. Using Poisson regression^16^, we performed bivariate analysis to estimate the prevalence ratio (PR) of susceptibility to measles infection (antibody titers ≤ 120 mIU/mL) by age, income, area of residence, crowding in the household and vaccination status. The analysis was carried out in the overall population and the subgroup of 1- to 5-year-old children. We conducted Poisson regression models for susceptibility to measles infection, adjusting for pertinent covariates for the total population (Model 1) and 1- to 5-year-old children (Model 2). We included in the models all statistically significant (p-values < 0.2), biologically plausible and relevant variables. Backward elimination procedure was used to create a more parsimonious model of variables that independently predicted susceptibility. The fitness of the model was checked by using the Hosmer–Lemeshow goodness-of-fit test. We selected the model with the best goodness of fit. We expressed the results as adjusted prevalence ratios (aPRs) with their corresponding 95% CIs. Prevalence estimates and regression models considered sampling weights using survey data commands in the statistical package STATA® 13.1.

### Ethics approval and informed consent

The Committees of Ethics, Biosafety and Research of the Instituto Nacional de Salud Pública approved the ENSANUT protocol and the specific protocol for the analysis of the serum samples. Informed consent/assent was obtained from the parents/guardians of children and from the adolescent and adult participants. The collection and management of data were carried out under confidentiality clauses according to Mexican regulations. We performed all methods according to these relevant guidelines and regulations.

## Results

We analyzed 7,785 blood samples. Of these, 7,721 had protective antibody titers (> 120 mIU/mL), which corresponded to a weighted seroprevalence of 99.37% (95% CI 99.07–99.58). Therefore, we estimate that approximately 92,773,400 Mexicans are protected against measles. The weighted susceptibility was 0.63% (95% CI 0.42–0.93), indicating that approximately 584,504 individuals are not protected against the disease.

Except for infants younger than 15 months, all age groups had a prevalence of measles antibodies greater than 96%.

**Figure 1:**
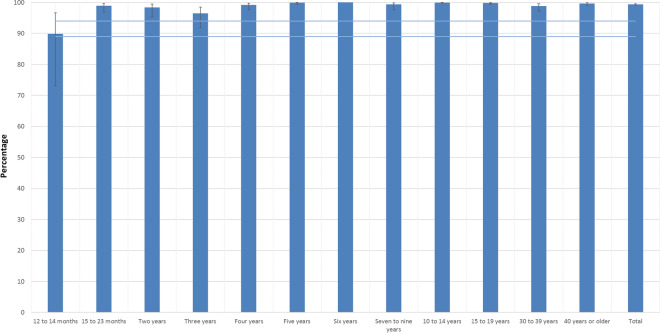
Percentage of seropositivity (PRNT antibodies > 120 mIU/mL) according to age group. We have highlighted the 89% and 94% cut-offs for achieving herd immunity.

As shown in Table 1, the age group with the highest susceptibility was the 12- to 14-month-old group (10.11%, 95% CI 3.32–26.89). At age 5 years, the susceptible proportion of the population decreased to 0.09% CI 0.01–0.63. At age 6 years, there were no susceptible children. We observed increased susceptibility among young adults who were 30–39 years old (1.17%, 95% CI 0.47–2.85). Among individuals aged 40 years and above, susceptibility was low (0.35%, 95% CI 0.14–0.91). In the 12- to 14-month-old, 3-year-old, 6-year-old and 10- to 14-year-old groups, the PR of antibody titers ≤ 120 mIU/mL was significantly different from that in the 40 years old or older age group (Table 1). Figure 1 shows the prevalence of PRNT antibodies according to age group. We have highlighted the 89% and 94% cut-offs for achieving herd immunity (Fig. 1).

**Table 1:** Prevalence of protective (> 120 mIU/mL) and nonprotective (≤ 120 mIU/mL) antibody titers by age. ENSANUT, 2012.

Age groups	Total No	Weighted total population	Antibody titers > 120 mIU/mL				Antibody titers ≤ 120 mIU/mL				PR	95% CI	p value*
			No	Weighted population	WP	95% CI	No	Weighted population	WP	95% CI			
12–14 months	39	418,255	33	375,968	89.89	73.11–96.68	6	42,287	10.11	3.32–26.89	28.81	6.90–120.28	< 0.001
15–23 months	150	1,266,771	145	1,252,893	98.90	96.81–99.63	5	13,878	1.10	0.37–3.19	3.12	0.74–13.14	0.120
2 years	282	2,683,081	277	2,638,950	98.36	95.29–99.44	5	44,131	1.64	0.56–4.71	4.69	1.12–19.57	0.034
3 years	372	2,101,642	364	2,026,804	96.44	91.92–98.47	8	74,838	3.56	1.53–8.08	10.15	2.85–36.10	< 0.001
4 years	368	2,489,869	362	2,469,289	99.17	97.62–99.72	6	20,580	0.83	0.28–2.38	2.36	0.56–9.85	0.240
5 years	163	2,228,312	162	2,226,324	99.91	99.37–99.99	1	1,987	0.09	0.01–0.63	0.25	0.03–2.24	0.217
6 years	180	2,296,598	180	2,296,598	100	–	0	0	0	–	0.00	–	< 0.001
7–9 years	669	6,972,506	666	6,925,667	99.33	97.63–99.81	3	46,839	0.67	0.19–2.37	1.91	0.39–9.36	0.422
10–14 years	1,470	11,272,359	1,466	11,265,317	99.94	99.82–99.98	4	7,042	0.06	0.02–0.18	0.18	0.04–0.74	0.017
15–19 years	1,260	11,323,493	1,254	11,298,072	99.78	99.43–99.91	6	25,422	0.22	0.09–0.57	0.64	0.17–2.41	0.509
30–39 years	811	16,079,550	798	15,892,150	98.83	97.15–99.53	13	187,400	1.17	0.47–2.85	3.32	0.90—12.29	0.072
40 years or older	2,021	34,225,467	2,014	34,105,368	99.65	99.09–99.86	7	120,100	0.35	0.14–0.91	Ref	–	–
Total	7,785	93,357,903	7,721	92,773,400	99.37	99.07–99.58	64	584,504	0.63	0.42–0.93	–	–	–

WP = weighted prevalence per 100 individuals; No = number; 95% CI, 95% confidence interval; PR, prevalence ratio; * p value for univariate Poisson regression.

Table 2 shows the sociodemographic and clinical variables of children, adolescents and adults stratified by the presence of protective titers. Belonging to the 1- to 4-year-old age group (PR 6.23, 95% CI 2.15–18.02; p = 0.001) and the 10- to 14-year-old age group (PR 0.18, 95% CI 0.04–0.74; p = 0.017) compared to the age group 40 years old or older was significantly associated with susceptibility. People living in crowded households were also more likely to be susceptible.

**Table 2:** Sociodemographic characteristics of the study population with and without protective titers. ENSANUT, 2012.

Characteristics	Total No	Weighted total population	Antibody titers > 120 mIU/mL				Antibody titers ≤ 120 mIU/mL						p-value*
			No	Weighted population	WP	95% CI	No	Weighted population	WP	95% CI	PR	95% CI	
**Sex**													
Male	3,614	44,257,092	3,585	44,027,186	99.48	99.09–99.70	29	229,905	0.52	0.30–0.91	Ref	–	–
Female	4,171	49,100,811	4,136	48,746,213	99.28	98.76–99.58	35	354,598	0.72	0.42–1.24	1.39	3.06	0.412
Total	7,785	93,357,903	7,721	92,773,400	99.37	99.07–99.58	64	584,504	0.63	0.42–0.93	–	–	–
**Age groups (years)**													
One to four	1,211	8,959,618	1,181	8,763,904	97.82	96.52–98.64	30	195,714	2.18	1.36–3.48	6.23	2.15–18.02	0.001
Five to nine	1,012	11,497,416	1,008	11,448,590	99.58	98.56–99.88	4	48,826	0.42	0.12–1.44	1.21	0.26–5.69	0.809
10 to 14	1,470	11,272,359	1,466	11,265,317	99.94	99.82–99.98	4	7,042	0.06	0.02–0.18	0.18	0.04–0.74	0.017
15 to 19	1,260	11,323,493	1,254	11,298,072	99.78	99.43–99.91	6	25,422	0.22	0.09–0.57	0.64	0.17–2.41	0.509
30 to 39	811	16,079,550	798	15,892,150	98.83	97.15–99.53	13	187,400	1.17	0.47–2.85	3.32	0.90–12.29	0.072
40 or more	2,021	34,225,467	2,014	34,105,368	99.65	99.09–99.86	7	120,100	0.35	0.14–0.91	Ref	–	–
**Crowded household**													
No	3,990	52,527,499	3,975	52,399,783	99.76	99.51–99.88	14	127,716	0.24	0.12–0.49	Ref	–	–
Yes	3,798	40,829,405	3,745	40,372,617	98.88	98.22–99.30	50	456,788	1.12	0.70–1.78	4.60	1.98–10.71	< 0.001

WP = weighted prevalence per 100 individuals; No = number; 95% CI, 95% confidence interval; PR, prevalence ratio.

* p value for univariate Poisson regression.

Supplementary Table S1 shows the sociodemographic and clinical variables of 1- to 5-year-old children with and without protective titers. Belonging to the 3-year-old group (PR 39.92, 95% CI 4.72–337.50; p = 0.001), the 2-year-old group (PR 18.44, 95% CI 1.99–170.74, p = 0.010), and the 1-year-old group (PR 37.37, 95% CI 4.57–305.76; p = 0.001) compared to the 5-year-old group was significantly associated with susceptibility. Children who had not been vaccinated (PR 7.82, 95% CI 2.94–20.83; p < 0.001) or who lived in crowded households (PR 7.80, 95% CI 2.08–29.34; p < 0.001) were more likely to be susceptible. Children living in Mexico City were less likely to be susceptible.

To identify variables associated with susceptibility to measles infection, we built two multivariate models: Model 1 was for children, adolescents and adults, and Model 2 was for 1- to 5-year-old children. In Model 1, the statistically significant variables associated with susceptibility were belonging to the 1- to 4-year-old age group (aPR 3.71, 95% CI 1.13–12.21; p = 0.031) and the 10- to 14-year-old age group (aPR 0.12, 95% CI 0.03–0.0.54; p = 0.006) compared to the age group 40 years old and older and living in a crowded household (aPR 3.65, 95% CI 1.43–9.27; p = 0.007). In Model 2, the variables associated with susceptibility among 1- to 5-year-old children were belonging to the 1-year-old age group (aPR 50.27, 95% CI 12.71–198.83; p < 0.001), 2-year-old age group (aPR 32.59, 95% CI 6.58–161.28; p < 0.001), 3-year-old age group (aPR 50.81%, 95% CI 12.97–198.96; p < 0.001), and 4-year-old age group (aPR 16.93%, 95% CI 3.70–77.35; p < 0.001) compared to the 5-year-old age group; living in a crowded household (aPR 6.83, 95% CI 1.72–27.16; p = 0.010); not having been vaccinated (aPR 6.63, 95% CI 2.65–14.05; p = 0.012); and unknown vaccination status (aPR 5.71. 95% CI 1.23–18.52, p = 0.012) (Table 3). Children living in Mexico City were less likely to be susceptible.

**Table 3:** Variables associated with titers ≤ 120 mIU/mL by multivariate analyses.

Characteristics	Crude analysis			Adjusted analysis		
	PR	95% CI	p-value*	aPR	95% CI	p-value**
	**All ages**					
**Age groups (years)**						
One to four	6.23	2.15–18.02	0.001	3.71	1.13–12.21	0.031
Five to nine	1.21	0.26–5.69	0.809	0.76	0.14–4.03	0.746
10 to 14	0.18	0.04–0.74	0.017	0.12	0.03–0.54	0.006
15 to 19	0.64	0.17–2.41	0.509	0.50	0.13–2.02	0.332
30 to 39	3.32	0.90–12.29	0.072	2.41	0.58–9.97	0.223
40 or more	Ref	–	–	Ref	–	–
**Household income (quintiles)**						
One (highest income)	Ref	–	–	Ref	–	–
Two	1.34	0.26–6.86	0.727	0.99	0.18–5.32	0.990
Three	1.24	0.27–5.61	0.784	0.82	0.17–4.00	0.802
Four	2.53	0.62–10.32	0.197	1.35	0.29–6.22	0.700
Five (lowest income)	2.55	0.63–10.25	0.187	1.01	0.22–4.71	0.991
**Zone**						
Central	Ref	–	–	Ref	–	–
Mexico City	0.91	0.11–7.60	0.927	0.99	0.10–9.51	0.991
Northern	2.39	0.92–6.23	0.074	2.43	0.94–6.27	0.066
Southern	2.06	0.75–5.61	0.158	1.84	0.70–4.80	0.215
**Crowded household**						
No	Ref	–	–	Ref	–	–
Yes	4.60	1.98–10.71	0.000	3.65	1.43–9.27	0.007
	**Children one-to-five-years old**					
**Age groups (years)**						
One	37.37	4.57–305.76	0.001	50.27	12.71–198.83	< 0.001
Two	18.44	1.99–170.74	0.010	32.59	6.58–161.28	< 0.001
Three	39.92	4.72–337.50	0.001	50.81	12.97–198.96	< 0.001
Four	9.27	0.99–86.66	0.051	16.93	3.70–77.35	< 0.001
Five	Ref	–	–	Ref	–	–
**Zone**						
Central	Ref	–	–	Ref	–	–
Mexico City	3.96e-10	1.57e-10–1.00e-09	< 0.001	7.09e-11	1.36e-11—3.68e-10	< 0.001
Northern	3.29	0.99–10.87	0.051	2.00	0.63–6.37	0.243
Southern	2.55	0.90–7.29	0.079	1.62	0.55–4.80	0.384
**Crowded household**						
No	Ref	–	–	Ref	–	–
Yes	7.80	2.08–29.34	< 0.001	6.83	1.72–27.16	0.010
**Vaccination status**						
Vaccinated	Ref	–	–	Ref	–	–
Unvaccinated	7.82	2.94–20.83	0.000	6.63	2.65–14.05	< 0.001
Unknown	0.20	0.03–1.57	0.125	5.71	1.23–18.52	0.012

WP = Weighted prevalence per 100 individuals; No = number; 95% CI, 95% confidence interval; PR, prevalence ratio; * p value for univariate Poisson regression; aPR, adjusted prevalence ratio; ** p value for multivariate Poisson regression; Ref, reference.

## Discussion

Determining the presence of specific antibodies against vaccine-preventable diseases is one of the most precise ways of evaluating immunization programs on the path towards elimination^17^.

Serosurveillance is used to identify areas of opportunity, eliminate the risk of outbreaks and guarantee the control, elimination and eradication of vaccine-preventable diseases^17,18^. As WHO regions advance towards measles elimination goals, there is an increasing need for evidence to inform risk and policy analyses. Previous models have used serosurveillance data to estimate the population immunity thresholds required to achieve elimination. Measles herd immunity levels vary according to setting and have been estimated to range between 89 to 94%^19^. Although our results showed that in general, susceptibility to measles was low overall (0.63%, 95% CI 0.42–0.93), serosusceptibility among children aged 1–4 years was considerable (2.18%, 95% CI 1.36–3.48). Of particular concern were children aged 12 to 14 months old, among whom 10.11% (95% CI 3.32–26.89) were serosusceptible. There may be several explanations for this finding, such as delays in receiving the first dose of the MMR vaccine, the failure to receive the vaccine or primary vaccine failure^20^. The likelihood of susceptibility was higher among nonvaccinated children than among vaccinated children, indicating that a programmatic failure to vaccinate was most likely the cause of the lack of antibodies. The ENSANUT 2012 results led to the estimation that approximately 195,714 1- to 4-year-old children were susceptible nationwide. Most susceptible children were between 12 and 14 months of age (the estimated number of susceptible 12- to 14-month-old children was approximately 42,287).

As age increased, we observed that susceptibility decreased. We found no susceptible children among 6-year-old individuals, which is the age at which the second dose of the measles vaccine is administered. Susceptibility increased among 30- to 39-year-old adults (1.17%, 95% CI 0.47–2.85) despite the MR vaccine having been recommended to individuals between 10 and 39 years of age who had not been vaccinated or who had an incomplete vaccination schedule^8^. Increased susceptibility among 30- to 39-year-old adults could be a birth cohort effect. The time frame in which these people were born (1973–1982) was during the early implementation phase of measles vaccination in Mexico, when high coverage levels had not yet been achieved, although the incidence of measles was decreasing. Therefore, a proportion of individuals from this birth cohort may have neither had the measles nor received the measles vaccine. Cruz-Hervert et al. reported that only 53.2% (95% CI 50.4–56.1) of women of reproductive age interviewed during this same survey had documented measles and rubella vaccination^21^. Most likely, susceptibility among 30- to 39-year-old persons reflects a combination of waning antibodies among individuals with incomplete vaccination schedules; the lack of antibodies among individuals who went unvaccinated during childhood, adolescence and young adulthood; and a lack of exposure to the naturally circulating virus (because the last endemic case occurred in 1995, almost 15 years before the present survey). Among individuals aged 40 years and older, susceptibility decreased to 0.35% (95% CI 0.14–0.91). This age range spans several decades, including people who were very likely infected during childhood. Similar trends have been reported in other studies^22,23^.

When compared to a previous survey with an analogous methodology, we observed similar measles seroprevalence levels among 1- to 4-year-old children, with values of 97.82% (95% CI 96.52–98.64) in our study and 98.30% (95% CI 97.7–98.7) in the study reported in 2000^14^.

Our results show that measles seroprevalence exhibits geographic variability, indicating that there may be spatial and socioeconomic determinants that may differentially impact vaccination coverage and hesitancy about vaccination, which merits further investigation. When analyzing children who were 1–5 years old, we found that individuals living in Mexico City were less likely to be susceptible than those living elsewhere. We consider that this lower susceptibility may be due to a low degree of marginalization and thus better access to health services^24,25^. A recent review concluded that vaccine hesitancy is a complex and context-specific phenomenon, fluctuating across space and time and depending on the specific vaccine^26^. Barriers to vaccination for those from a low socioeconomic background include, among other circumstances, unemployment, lack of access to health services, neighborhood poverty, migration patterns, a female head-of-household, and a lower education level^27^. In contrast, the barriers for those of a higher socioeconomic level may be more likely due to anti-vaccination propaganda circulated on the internet and social media^28^.

One of the main milestones on the path towards the global elimination of measles established by the World Health Assembly was an increase in routine coverage with the first dose of measles-containing vaccine for children older than 1 year to more than 90% nationally and more than 80% in every district. The region of the Americas achieved the elimination of measles in 2016. However, this region lost this status by 2018. Measles elimination goals have not been achieved because immunization coverage gaps persist due to weak and fragile health systems, civil unrest, famine, active conflict, vaccine hesitancy and declining maternal antibody levels^29,30^. Additional challenges include susceptible persons being distributed across increasingly broad age groups, making elimination more expensive and more technically demanding. Consequently, outbreaks affect older age groups (adolescents and adults), different communities (migrants, religious groups), and infants younger than 1 year of age. Therefore, the region of the Americas faces a challenge with regard to reattaining the elimination of measles, with ongoing outbreaks and the resurgence of the disease in other areas. More than 5,000 measles cases were recorded between 2011 and 2018 in Brazil, Canada, Ecuador, the United States, and Venezuela, among other countries^31^. The importation of measles from endemic areas continues to occur^32,33^. In Mexico, outbreaks started by these imported cases are short-lived, most likely due to herd immunity among age groups with the highest transmission rates, as suggested by Al-Mazrou et al^34^.

This study's limitations include its cross-sectional design, which constrained our ability to make causal inferences. The main strength of this study was the usage of PRNT titers, which are the best correlate of protection. Additional advantages were its large sample size, which allowed us to evaluate possible confounders in statistical models, as well as its sampling framework, which allowed the weighted estimation of the seroprevalence and serosusceptibility among different subgroups. The design of the survey anticipated response rates between 78.3% and 81.0%. Given that the response rate in our study was 87%, which was higher than the expected rate, there is only a slight potential for bias due to nonparticipation.

This study demonstrates that Mexico maintains high levels of protection against measles across nearly all age groups, thus explaining the lack of endemic transmission. However, variables associated with susceptibility were young age, unvaccinated status, living in specific geographical areas, and living in crowded conditions. Among susceptible groups, it is essential to ensure vaccination in 12-month-old children. As measles transmission persists in many countries, it is necessary to maintain vaccine coverage above 95% with two doses of a measles-containing vaccine to prevent outbreaks due to imported cases.

## Supplementary information

Supplementary information.

## Data Availability

All data generated or analyzed during this study are included in this published article.
