# Supplementary material for: Seroprevalence of measles antibodies and factors associated with susceptibility: a national survey in Mexico using a plaque reduction neutralization test
Source: Sci Rep. 2020 Oct 15;10:17488. doi: 10.1038/s41598-020-73618-8 (PMC7562930; doi:10.1038/s41598-020-73618-8)
Supplement: Supplementary file 1 — Supplementary information. [file 41598_2020_73618_MOESM1_ESM.pdf]

Seroprevalence of measles antibodies and factors associated with susceptibility: a national survey in Mexico using a plaque reduction neutralization test.

José Luis Díaz-Ortega, Elizabeth Ferreira-Guerrero, Luis Pablo Cruz-Hervert, Guadalupe Delgado-Sánchez, Leticia Ferreyra-Reyes, Mercedes Yanes-Lane, Norma Mongua-Rodríguez, Rogelio Montero-Campos, Deyanira Castañeda-Desales, Lourdes Garcia-Garcia*

Supplementary Table S1. Sociodemographic characteristics of study children from one to five years of age with and without protective titers. ENSANUT, 2012

Characteristics	Total No.	Weighted total population	Antibody titers > 120 mUI/mL				Antibody titers ≤120 mUI/mL				PR	95% CI	p-value*
			No.	Weighted population	WP	95% CI	No.	Weighted population	WP	95% CI			
**Sex**													
Male	689	5,684,773	672	5,569,112	97.97	96.15-98.94	17	115,662	2.03	1.06-3.85	Ref.	-----	-----
Female	685	5,503,156	671	5,421,116	98.51	97.12-99.23	14	82,040	1.49	0.77-2.88	0.73	0.29-1.86	0.513
Total	1,374	11,187,929	1,343	10,990,228	98.23	97.20-98.89	31	197,701	1.77	1.11-2.80	-----	-----	-----
**Age groups (years)**													
One	189	1,685,026	178	1,628,861	96.67	93.18-98.40	11	56,165	3.33	1.60-6.82	37.37	4.57-305.76	0.001
Two	282	2,683,081	277	2,638,950	98.36	95.28-99.44	5	44,131	1.64	0.56-4.72	18.44	1.99-170.74	0.010
Three	372	2,101,642	364	2,026,804	96.44	91.90-98.48	8	74,838	3.56	1.52-8.10	39.92	4.72-337.50	0.001
Four	368	2,489,869	362	2,469,289	99.17	97.61-99.72	6	20,580	0.83	0.28-2.39	9.27	0.99-86.66	0.051
Five	163	2,228,312	162	2,226,324	99.91	99.37-99.99	1	1,987	0.09	0.01-0.63	Ref.	-----	-----
**Vaccination status**													
Vaccinated	1,176	9,230,759	1,158	9,127,806	98.88	97.94-99.40	18	102,953	1.12	0.60-2.06	Ref.	-----	-----

Unvaccinated	124	1,062,965	112	970,204	91.27	82.24-95.94	12	92,761	8.73	4.06-17.76	7.82	2.94-20.83	<0.001
Unknown	74	894,205	73	892,217	99.78	98.42-99.97	1	1,987	0.22	0.03-1.58	0.20	0.03-1.57	0.125
**Household income (quintiles)**													
1 (highest income)	679	967,150	675	946,527	99.67	98.89-99.90	4	20,623	0.33	0.10-1.11	Ref.	-----	-----
2	1,171	1,564,659	1,165	1,560,558	99.56	98.68-99.85	6	4,101	0.44	0.15-1.32	0.12	0.01-1.87	0.131
3	1,358	2,068,270	1,351	2,033,22	99.59	99.00-99.83	7	35,044	0.41	0.17-1.00	0.79	0.08-8.17	0.846
4	1,683	1,917,526	1,666	1,877,713	99.16	98.26-99.60	17	39,813	0.84	0.40-1.74	0.97	0.13-7.35	0.979
5 (lowest income)	2,893	4,670,326	2,863	4,572,204	99.15	98.32-99.57	30	98,121	0.85	0.43-1.68	0.99	0.14-7.02	0.988
**Zone**													
Central	496	4,645,807	487	4,601,897	99.05	97.90-99.58	9	43,911	0.95	0.42-2.10	Ref.		
Mexico City	19	786,479	19	786,479	100	-----	0	0	0	-----	3.96E-10	1.57e-10-1.00e-09	<0.001
Northern	252	2,141,353	246	2,074,842	96.89	92.58-98.73	6	66,511	3.11	1.27-7.42	3.29	0.99-10.87	0.050
Southern	607	3,614,289	591	3,527,010	97.59	95.29-98.78	16	87,279	2.41	1.22-4.71	2.55	0.90-7.29	0.080
**Location**													
Urban	778	8,341,858	759	8,198,862	98.29	96.98-	19	142,996	1.71	0.97-3.02	1.12	0.44-2.86	0.810

						99.03						
Rural	596	2,846,072	584	2,791,366	98.08	95.99-99.09	12	54,705	1.92	0.91-4.01	Ref.	-----	-----
**Access to social security**													
Yes	1,083	8,262,350	1,064	8,148,584	98.62	97.57-99.22	19	113,766	1.38	0.78-2.43	Ref.	-----	-----
No	289	2,920,469	277	2,836,534	97.13	93.83-98.69	12	83,935	2.87	1.31-6.17	2.09	0.80-5.46	0.134
**Speaks indigenous language**													
No	830	6,473,188	816	6,376,866	98.51	97.03-99.26	14	96,322	1.49	0.74-2.97	0.21	0.03-1.65	0.138
Yes	73	346,635	72	345,551	99.69	97.84-99.96	1	1,084	0.31	0.04-2.16	Ref.	-----	-----
**Crowded household**													
No	397	3,604,496	394	3,593,146	99.69	98.94-99.91	3	11,350	0.31	0.09-1.06	Ref.	-----	-----
Yes	977	7,583,433	949	7,397,081	97.54	96.01-98.50	28	186,352	2.46	1.50-3.99	7.80	2.08-29.34	<0.001

WP= weighted prevalence per 100 individuals; No.=number; 95% CI, 95% confidence interval; PR, prevalence ratio; * p value for univariate Poisson regression.
